# Comparative predictive value of AIMS65, Pre-endoscopic Rockall and Glasgow-Blatchford Scoring Systems in Nonvariceal Upper Gastrointestinal Bleeding

**DOI:** 10.12669/pjms.42.5.15131

**Published:** 2026-05

**Authors:** Tingting Cui, Zhongsheng Cao

**Affiliations:** 1 Tingting Cui, Department of Gastroenterology, The First People’s Hospital of Jiashan, Jiashan Hospital Affiliated to Jiaxing University, Jiaxing, Zhejiang Province 314100, P.R. China; 2 Zhongsheng Cao, Department of Gastroenterology, The First People’s Hospital of Jiashan, Jiashan Hospital Affiliated to Jiaxing University, Jiaxing, Zhejiang Province 314100, P.R. China

**Keywords:** AIMS65, Glasgow-Blatchford score, Non-variceal upper gastrointestinal bleeding, Predictive ability, Pre-endoscopic Rockall score

## Abstract

**Objective:**

To compare the predictive ability of the AIMS65, pre-endoscopic Rockall score (Pre-RS), and Glasgow-Blatchford score (GBS) for clinical outcomes in patients with non-variceal upper gastrointestinal bleeding (NVUGIB).

**Methodology:**

This retrospective, single-center, observational study analyzed the clinical records of 314 patients with NVUGIB who presented to Jiashan County First People’s Hospital between October 2023 and June 2025. The AIMS65 score, Pre-RS, and GBS were retrospectively calculated for these patients. The predictive ability of the scores for in-hospital mortality, transfusion requirement, endoscopic intervention, and rebleeding was evaluated by calculating the area under the receiver operating characteristic curve (AUC).

**Results:**

Of 314 patients included in the study, 9.6% died during hospitalization, 40.1% required blood transfusion, 76.4% underwent endoscopic intervention, and 6.1% experienced rebleeding. The AIMS65, Pre-RS, and GBS all demonstrated good performance in predicting the clinical endpoints. The AIMS65 showed significantly greater predictive ability for in-hospital mortality than Pre-RS and GBS. The GBS had a markedly superior ability to predict the need for blood transfusion compared with the other scores, and the Pre-RS exhibited a significantly better predictive performance for endoscopic intervention. No significant differences were observed among the three scores in terms of their predictive efficacy for rebleeding.

**Conclusions:**

The AIMS65, Pre-RS, and GBS demonstrated good predictive value for clinical endpoints in patients with NVUGIB. The AIMS65 was more effective in predicting in-hospital mortality, GBS showed the best performance for predicting transfusion requirements, and Pre-RS performed best for predicting endoscopic intervention. No significant difference was observed among the three scores in predicting rebleeding.

## INTRODUCTION

Acute non-variceal upper gastrointestinal bleeding (NVUGIB) is a common and critical emergency in gastroenterology and emergency departments, with an incidence of approximately 60–80 cases per 100,000 population annually, and a mortality rate of 2–10%.^1^ NVUGIB can lead to hemorrhagic shock, prolonged hospital stays, and is considered one of the important causes of death in elderly patients and those with multiple systemic comorbidities.^2^,^3^ Peptic ulcer remains the primary etiology of NVUGIB.^3^ However, with improvements in Helicobacter pylori eradication population aging, and the widespread use of antiplatelet and anticoagulant drugs, the proportion of drug-related mucosal injury and bleeding in elderly patients and those with cardiovascular and cerebrovascular diseases is on the rise.^1^,^3^ Therefore, accurate early risk stratification for NVUGIB patients is of great significance for guiding diagnosis and treatment strategies and optimizing resource allocation.

In recent years, the AIMS65 has emerged as a simplified pre-endoscopic risk stratification tool comprising only five readily measurable variables: serum albumin level, international normalized ratio (INR), mental status, systolic blood pressure, and patient age. Robertson et al.^4^ reported that the AIMS65 demonstrates robust predictive performance for in-hospital mortality, length of hospital stay, and hospitalization costs in patients with upper gastrointestinal bleeding. A head-to-head comparison of the AIMS65, Glasgow-Blatchford score (GBS), and pre-endoscopic Rockall score (Pre-RS) showed that the AIMS65 outperforms both the GBS and Pre-RS in predicting in-hospital mortality, matching the prognostic accuracy of the full Rockall score, and demonstrates superior performance in forecasting length of hospital stay and the need for intensive care unit (ICU) admission. In contrast, Bozkurt et al.^5^ compared the modified early warning score (MEWS), GBS, and Pre-RS in an emergency department cohort of patients with gastrointestinal bleeding and showed that the predictive efficacy of these scoring systems varies considerably across different clinical endpoints (including mortality, rebleeding, and requirement for invasive intervention). These discrepancies highlight the need to select the most appropriate risk assessment tool based on the specific clinical context and the targeted outcome of interest.

Large-scale prospective studies have further highlighted the heterogeneous performance of various risk scoring systems in upper gastrointestinal bleeding. Stanley et al.^6^ compared multiple risk scores in an international, multicenter cohort and concluded that GBS performed best overall in predicting the composite endpoint of “any clinical intervention or mortality”. In contrast, the AIMS65 and Progetto Nazionale Emorragia Digestive (PNED) scores demonstrated superior discriminative ability for mortality prediction. The CHAMPS score developed by Matsuhashi et al.^7^ outperformed the GBS, AIMS65, and clinical Rockall scores in predicting in-hospital mortality among patients with pure NVUGIB.

However, this score is limited by its extensive variable set, complex calculation process, and validation primarily in Japanese populations. Collectively, prior research has identified niche applications of each scoring system. The Rockall score retains a certain value in predicting rebleeding and mortality among elderly and high-risk populations.^8^ The GBS is better suited for identifying patients requiring clinical intervention or eligible for safe discharge,^6^ whereas the AIMS65 is advantageous for predicting mortality, ICU admission, and length of hospital stay.^4^ Nevertheless, marked heterogeneity in the performance and optimal cutoff values of these scores has been observed across studies, which can be attributed to discrepancies in endpoint definitions, study populations (such as inclusion of variceal bleeding cases, mixing of upper and lower gastrointestinal bleeding, and enrollment of inpatient vs. outpatient cohorts), healthcare systems, and therapeutic strategies.

Notably, most comparative studies have failed to conduct analyses specifically targeting NVUGIB, a relatively homogeneous etiological spectrum. Instead, some studies have included variceal bleeding or lower gastrointestinal bleeding cases in their cohorts. Furthermore, most study populations have been derived from Europe, North America, or Japan, resulting in a paucity of real-world data from other Asian regions, particularly China. Although Barkun et al.^9^ and subsequent clinical guidelines have repeatedly emphasized the routine application of risk scores during the emergency and early hospitalization phases, the utilization rate of these tools remains suboptimal in actual clinical practice, and no single scoring system has been universally accepted as the gold standard for NVUGIB management.^10^ For instance, in China, NVUGIB patients are predominantly elderly and burdened with multiple chronic comorbidities, with widespread use of antiplatelet and anticoagulant therapies. The favorable performance of the Rockall score in Chinese elderly NVUGIB patients suggests that risk-stratification strategies for local populations may have unique characteristics.^8^

Therefore, the present study aimed to systematically compare the predictive performance of AIMS65, Pre-RS, and GBS scores in a cohort of patients with NVUGIB to guide early clinical decision-making in the emergency department (ED). Specifically, we evaluated the accuracy of AIMS65 in identifying patients at high risk of mortality for intensive care triage, the utility of GBS in anticipating transfusion requirements for blood resource planning, and the effectiveness of Pre-RS in predicting the need for endoscopic intervention to prioritize therapy. By aligning these scores with specific clinical decision points, we aimed to provide evidence-based support for optimizing risk-stratification strategies tailored to the local clinical context.

## METHODOLOGY

This retrospective, single-center, observational study included the clinical records of consecutive patients suspected of NVUGIB presenting with vomiting blood, black stool, or decreased hemoglobin levels at the First People’s Hospital of Jiashan County between October 2023 and June 2025. All patients underwent emergency upper gastrointestinal endoscopy within 24 hours. The patients were followed up for 30 days after discharge.

### Ethical approval:

The ethics committee of the First People’s Hospital of Jiashan County approved this retrospective study with the number LW2025067; Date: November 3^rd^, 2025.

### Inclusion criteria:

- The patient presents with symptoms such as black stool, vomiting blood, rectal bleeding, positive vomit or fecal occult blood, and abdominal discomfort.
- Accompanied or not by peripheral circulatory disorders such as dizziness, palpitations, and cold sweats.
- Bleeding lesions found after endoscopic examination.
- Age ≥ 18 years old.

### Exclusion criteria

- Variceal bleeding.
- Bleeding from iatrogenic lesions after endoscopic resection.
- Unable to obtain at least one of the three rating systems.

To calculate the AIMS65, Pre-RS, and GBS, relevant clinical variables were extracted via manual chart review, including patient demographics, presenting symptoms, comorbidities, baseline laboratory values, endoscopic findings, and treatment details. All clinical and laboratory data utilized for scoring were collected during the patient’s initial evaluation upon admission (baseline). Laboratory markers were obtained from the first tests within two hours of arrival; vital signs (systolic blood pressure and heart rate) reflected the first recorded values at triage. Altered mental status was determined based on the initial clinical assessment upon admission or a Glasgow Coma Scale (GCS) score of <15.

Using receiver operating characteristic (ROC) curves, the utility of the three scoring systems in predicting in-hospital mortality, transfusion requirements, endoscopic intervention, and rebleeding was compared. In-hospital mortality included deaths both related and unrelated to NVUGIB. Rebleeding was defined as a daily decrease in hemoglobin concentration of at least 2.0 g/dL, accompanied by repeated endoscopic examinations due to unstable vital signs, before discharge, surgery, transarterial embolization for upper gastrointestinal bleeding (UGIB) control, or readmission within 30 days after discharge for UGIB. Patient management followed a standardized, guideline-based protocol. Prior to emergency endoscopy, patients typically received intravenous proton pump inhibitors (PPIs) and fluid resuscitation based on their hemodynamic status. Transfusion requirements strictly followed established criteria (Hb < 7 g/dL for general patients and < 8 g/dL for high-risk cardiac patients). While care generally adhered to these standards, specific interventions remained at the discretion of the attending clinician based on individual clinical judgment. Endoscopic interventions encompassed epinephrine injection, hemostatic electrocoagulation, argon plasma coagulation, or endoscopic clip application. In cases of failed endoscopic hemostasis, transarterial embolization was preferred over surgical intervention. When rebleeding occurred after successful endoscopic treatment, endoscopic therapy was initially the first-line option; surgery was performed as salvage therapy if embolization failed.

### Statistical analysis:

Data analyses were performed using IBM SPSS Statistics software (Version 26.0, SPSS Inc., Chicago, IL, USA). Categorical variables were described as percentages. The normality of continuous variables was assessed visually (histograms and Q-Q plots) and analytically (Kolmogorov-Smirnov and Shapiro-Wilk tests). Non-normally distributed data were presented as medians with interquartile ranges (IQR). To quantify the predictive discriminative ability of each scoring system, the area under the receiver operating characteristic curve (AUC) and its corresponding 95% confidence intervals (CIs) were calculated using a non-parametric approach. ROC curve analyses were performed utilizing IBM SPSS Statistics, and the pairwise comparison of AUCs across the three scoring systems was evaluated using DeLong’s test. Given the exploratory and comparative nature of this study involving multiple clinical outcomes and score comparisons, we adopted a cautious approach in interpreting marginal statistical findings without applying formal adjustments for multiplicity. Additionally, the sensitivity and specificity (with 95% CIs) of the AIMS65, Pre-RS, and GBS were computed for predicting in-hospital mortality, transfusion requirement, need for endoscopic intervention, and rebleeding, respectively. A two-sided p-value < 0.05 was considered statistically significant.

## RESULTS

The demographic and clinical characteristics, treatment, and outcomes of the participants are shown in Table-I. Of the patients initially screened for suspected bleeding, 107 were excluded. Among them, 55 were excluded for not meeting the definition of NVUGIB (including 28 cases of lower gastrointestinal bleeding, 19 cases of variceal bleeding, and eight cases of anemia due to non-bleeding causes); 43 were excluded due to incomplete laboratory data, and nine were excluded due to loss to follow-up. Finally, a total of 314 patients with NVUGIB were included (198 (63.1%) males and 116 (36.9%) females). The median age of patients was 62 years (range 18-92). The most common symptom at admission was black stool, with 186 cases (59.2%). Among the patients, 249 (79.3%) had comorbidities. The common causes of bleeding were gastric ulcer (54.1%), duodenal ulcer (23.9%), Mallory Weiss tear (11.5%), and malignant tumor (3.8%). Thirty patients (9.6%) died during hospitalization, 126 patients (40.1%) required blood transfusion, 240 patients (76.4%) required endoscopic intervention, and 19 patients (6.1%) experienced rebleeding.

**Table-I:** Basic characteristics of patients.

Characteristic	Value
Age (year), median(IQR)	62 (54-71)
***Sex, n(%)***	
Male	198 (63.1)
Female	116 (36.9)
***Clinical variables, n(%)***	
Hematemesis	42 (13.4)
Hematemesis and melena	57 (18.2)
Melena	186 (59.2)
Melena and syncope	21 (6.7)
Hematemesis, melena and syncope	8 (2.5)
***Comorbidity, n(%)***	
None	65 (20.7)
Hypertension	151 (48.1)
Diabetes mellitus	58 (18.5)
Liver disease	24 (7.6)
Chronic renal impairment	22 (7.0)
Ischemic heart disease	51 (16.2)
Metastatic cancer	22 (7.0)
Chronic obstructive airways disease	16 (5.1)
***Lab variables, median(IQR)***	
Hemoglobin (g/L)	96.5 (76-124)
Albumin (g/L)	34.6 (31.9-36.7)
International normalized ratio (unitless)	1.05 (0.96-1.13)
Blood urea nitrogen (mmol/L)	7.82 (4.67-12.19)
Serum creatinine (μmol/L)	94 (72-125)
***Bleeding cause of endoscopic finding, n(%)***	
Gastric ulcer	170 (54.1)
Duodenal ulcer	75 (23.9)
Mallory Weiss tear	36 (11.5)
Malignancy	12 (3.8)
Dieulafoy’s lesion	8 (2.5)
Esophageal ulcer	6 (1.9)
Others	7 (2.2)
***Clinical outcomes, n(%)***	
In-hospital mortality	30 (9.6)
Transfusion requirement	126 (40.1)
Endoscopic intervention	240 (76.4)
Rebleeding	19 (6.1)

***Note:*** Data are presented as median (interquartile range) for continuous variables, and as frequency (percentage) for categorical variables.

**Fig.1:**
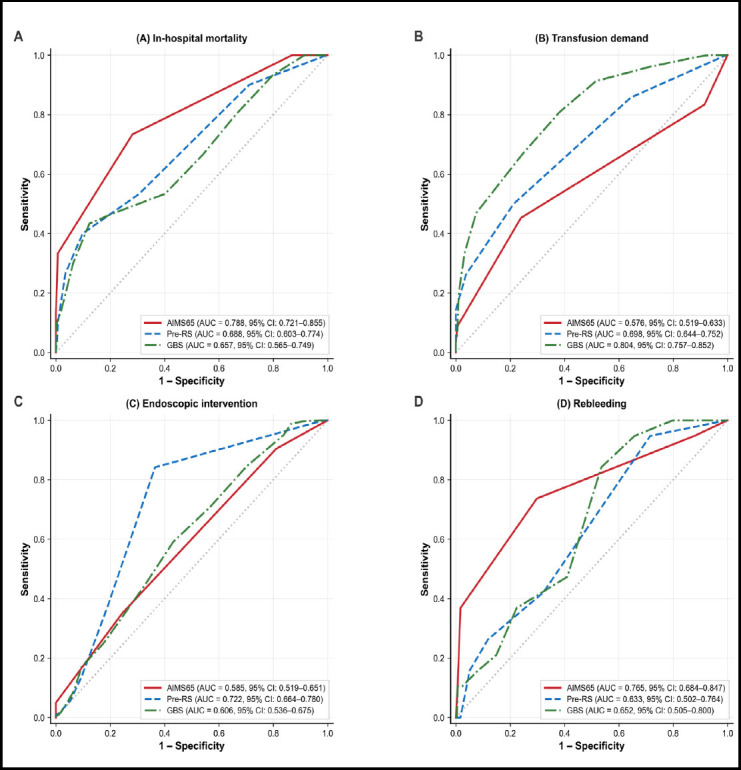
ROC curves of the AIMS65, Pre-RS, and GBS for predicting clinical endpoints. (A) Used to predict in-hospital mortality rate, (B) Transfusion demand, (C) Endoscopic intervention and (D) Rebleeding.

The AUCs for predicting in-hospital mortality, transfusion demand, endoscopic intervention, and rebleeding scores are shown in Fig.1, and the comparison of AUCs is summarized in Table-II. For predicting in-hospital mortality, AIMS65 demonstrated significantly higher predictive value (AUC=0.788) than Pre-RS and GBS. Clinically, the improvement of the AUC from 0.657 (GBS) to 0.788 (AIMS65) directly translates into a lower rate of missed high-risk cases, allowing clinicians to initiate intensive care triage more promptly. For transfusion demand, GBS (AUC=0.804) was markedly superior, assisting clinicians in more accurately identifying low-risk patients for safe discharge, thereby optimizing blood resource allocation. Regarding endoscopic intervention, Pre-RS (AUC=0.722) showed the best predictive performance. Finally, although AIMS65 showed a numerically higher AUC for rebleeding, no statistically significant differences were observed among the three scores.

**Table-II:** Comparison of AUC under ROC curve for predicting clinical endpoints using AIMS65, Pre-RS, and GBS scores.

	AUC (95% CI)	P-value of paired AUC curves		
		AIMS65	Pre-RS	GBS
***In-hospital mortality (n=30)***				
AIMS65	0.788 (0.721–0.855)	-	0.031	0.002
Pre-RS	0.688 (0.603–0.774)	-	-	0.440
GBS	0.657 (0.565–0.749)	-	-	-
***Transfusion requirement (n=126)***				
AIMS65	0.576 (0.519–0.633)	-	0.002	<0.001
Pre-RS	0.698 (0.644–0.752)	-	-	0.001
GBS	0.804 (0.757–0.852)	-	-	-
***Endoscopic intervention (n=240)***				
AIMS65	0.585 (0.519-0.651)	-	0.003	0.640
Pre-RS	0.722 (0.664-0.780)	-	-	0.003
GBS	0.606 (0.536-0.675)	-	-	-
***Rebleeding (n=19)***				
AIMS65	0.765 (0.684-0.847)	-	0.092	0.054
Pre-RS	0.633 (0.502-0.764)	-	-	0.709
GBS	0.652 (0.505-0.800)	-	-	-

***Note:*** AUC: Area under the receiver operating characteristic curve; CI: Confidence interval; Pre-RS: Pre-endoscopic Rockall score; GBS: Glasgow-Blatchford score.

## DISCUSSION

This study compared the predictive value of the AIMS65, Pre-RS, and GBS scores in patients with NVUGIB. Overall, AIMS65 demonstrated robust predictive performance for identifying high in-hospital mortality risk, which is a relatively hard endpoint. In contrast, as process-driven indicators, the outcomes for transfusion and endoscopic intervention are influenced to some extent by clinical pathways and resource accessibility. Our results indicate that GBS and Pre-RS performed better for identifying patients likely to require blood transfusion and early endoscopic intervention, respectively. Furthermore, for the composite endpoint of rebleeding, which is influenced by both physiological indicators and endoscopic hemostasis, the overall predictive performance of all three scoring systems was inherently moderate. This distinction underscores the necessity of selecting the appropriate scoring system based on specific clinical management goals, rather than using them as the sole determinant for treatment protocols.

These findings support the notion that different scoring tools reflect distinct dimensions of bleeding-related risk, and to date, no single “universal” scoring system exists that can accurately predict all clinical endpoints.^11^–^13^

In terms of mortality risk prediction, this study found that AIMS65 was significantly superior to both Pre-RS and GBS, which is consistent with previous reports. A Korean NVUGIB cohort study by Kim et al.^14^ showed that the AUC of the AIMS65 for in-hospital mortality was approximately 0.8 and was at least non-inferior to those of the GBS, Pre-RS, and full Rockall score, while also being easier to calculate. Similarly, Nasr Isfahani et al.^15^ found that among patients with upper gastrointestinal bleeding, the AIMS65 and Pre-RS had favorable discriminative ability for 30-day mortality, outperforming the GBS. A large, multicenter, prospective study by Stanley et al.^6^ and subsequent systematic reviews further confirmed that the AIMS65 was generally non-inferior to the Rockall and GBS scores for short-term mortality prediction. However, newly proposed scoring systems, such as the ABC score, have shown better performance in some studies.^11^,^12^,^16^,^17^ Integrating this body of evidence, the results of the present study support the use of the AIMS65 as the first-choice or key reference tool in clinical practice when the primary endpoint of interest is mortality or severe clinical outcomes.

The performance of the Pre-RS in this study also merits discussion. The Rockall scoring system was originally developed to predict mortality and rebleeding risk, whereas the Pre-RS represents its pre-endoscopic component and can be applied before endoscopic findings are available..^6^,^8^,^18^ Kaminskis et al.^19^ reported that among elderly NVUGIB patients, a Rockall score ≥7 could identify a subgroup at high risk of rebleeding; prophylactic transarterial embolization in this population significantly reduced rebleeding incidence and the need for surgical intervention. However, the requirement for endoscopic findings to calculate the full Rockall score limits its utility in ultra-early risk stratification in the emergency setting. Work by Orpen-Palmer et al.^16^ and Stanley et al.^6^ has demonstrated that in current clinical practice, the Rockall score is less frequently used for initial risk stratification, with greater reliance placed on pre-endoscopic tools such as the AIMS65 and GBS scores. Consistent with these findings, the present study showed that Pre-RS had a particular advantage in predicting the need for endoscopic intervention, whereas AIMS65 and GBS performed better for mortality and transfusion prediction, respectively.

For composite outcomes including rebleeding, the need for endoscopic or interventional therapy, and ICU admission, the predictive ability of all three scoring systems in this study was limited, which echoes prior reports.^20^ In the study by Kim et al.,^14^ the AUC of ROC of the AIMS65, GBS, and Rockall scores for rebleeding all fell within the moderate range. Their predictive performance across different secondary endpoints was not entirely consistent. A systematic review and meta-analysis of pre-endoscopic scoring systems also revealed that most scores have limited accuracy in predicting the need for high-risk interventions. However, GBS has a slight advantage in identifying patients who require blood transfusion or endoscopic hemostasis.^17^ In the present study, the GBS demonstrated superior predictive performance for the need for transfusion and endoscopic intervention than for mortality, whereas the AIMS65 was more advantageous for mortality prediction. This pattern is highly consistent with the previous studies,^20^ suggesting that clinicians should selectively adopt scoring systems based on the specific clinical outcomes of interest in practice. However, given the limited number of rebleeding events and the absence of statistically significant differences among the three scores, it is crucial to emphasize that these scoring systems should complement rather than replace clinical judgment. They must be viewed primarily as adjunctive tools for early triage. The final management plan must still be dynamically adjusted based on the patient’s real-time clinical status, physiological monitoring, and professional clinician judgment.

Furthermore, recent research has demonstrated that early pre-endoscopic intervention with potent proton pump inhibitors, such as Eprazole Sodium combined with somatostatin, can significantly improve coagulation function in patients with upper gastrointestinal bleeding.^21^ Such pharmacological interventions may stabilize clot formation, thereby influencing the predictive performance of risk-scoring systems (particularly those incorporating coagulation markers like AIMS65) for rebleeding and mortality.

### Strength of the Study:

First and foremost, the cohort exclusively comprised patients with NVUGIB, thereby eliminating confounding from portal hypertension-related bleeding. This ensured a more homogeneous study population, consistent with the patient inclusion criteria of most clinical guidelines and risk score development studies.^9^,^10^,^22^ Second, all enrolled patients received current standard-of-care endoscopic and pharmacological therapies, and key clinical endpoints (including in-hospital mortality, rebleeding, blood transfusion, and endoscopic intervention) were systematically documented. This enabled a rigorous head-to-head comparison of AIMS65, Pre-RS, and GBS scores across multiple clinical outcomes. Third, beyond evaluating the discriminative ability of each scoring system, the study compared their performance across clinically relevant endpoints and proposed practical recommendations from the perspective of clinical decision-making. These findings are expected to facilitate the optimization of patient triage and resource allocation in endoscopy centers and emergency departments.

### Limitations:

First, the single-center, retrospective design introduces potential selection bias. Clinician variations in pre-endoscopic medications, resuscitation strategies, and transfusion thresholds may have influenced clinical outcomes. Furthermore, our ulcer-dominant cohort (>78%) and strict 24-hour endoscopy mandate mean that findings regarding endoscopic intervention prediction (such as the advantage of the Pre-RS) are highly context-dependent. Conversely, AIMS65’s mortality prediction remains a robust finding due to its focus on systemic physiology. Second, lacking the systematic 30-day follow-up seen in multicenter cohorts,^6^,^15^ we could not assess post-discharge outcomes. Third, we did not evaluate novel scoring systems (such as the ABC, CANUKA, and PNED scores) that might outperform traditional tools.^5^,^16^,^17^,^23^ Additionally, the limited number of events for mortality and rebleeding precluded in-depth subgroup sensitivity analyses; future large-scale studies are needed to validate the consistency of these AUC rankings. Finally, scores were only calculated at baseline, whereas dynamic reassessments incorporating post-resuscitation and endoscopic findings could further improve predictive accuracy.^16^,^24^

### Pragmatic clinical application recommendations:

Based on the results of this study, we recommend that clinicians adopt a multi-dimensional risk-stratification strategy: first, prioritize the AIMS65 score for early triage to identify patients at high risk for in-hospital mortality and ensure timely admission to intensive care units; second, utilize the GBS score to anticipate blood transfusion requirements, thereby optimizing blood resource allocation and resuscitation planning in the emergency department; finally, refer to the Pre-RS score to assess the urgency of endoscopic intervention and rationally prioritize procedural schedules. It is important to note that these scoring systems should supplement rather than replace clinical judgment, and their implementation should flexibly reflect local hospital workflows, resource availability, and individual patient clinical characteristics.

## CONCLUSION

While AIMS65, Pre-RS, and GBS all provide clinical value for NVUGIB, they serve distinct purposes. AIMS65 excels in mortality risk stratification, GBS is optimal for predicting transfusion needs, and Pre-RS effectively anticipates the need for endoscopic intervention. Given their limited efficacy in predicting rebleeding, these tools should complement comprehensive clinical judgment to optimize early triage and resource allocation.

### Author’s contributions:

**TC:** Literature search, study design and manuscript writing, manuscript revision, validation and is responsible for the integrity of the study.

**TC and ZC:** Data collection, data analysis and interpretation.

All authors have read and approved the final version of the manuscript.
